# Ants of Ambon Island – diversity survey and checklist

**DOI:** 10.3897/zookeys.472.8441

**Published:** 2015-01-19

**Authors:** Fransina Latumahina, Michaela Borovanska, Musyafa, Sumardi, Nugroho Susetya Putra, Milan Janda

**Affiliations:** 1 Faculty of Forestry, Gadjah Mada University, Jalan Bulaksumur, Yogyakarta, 55281, Indonesia; 2 Pattimura University, Jalan Ir M Putuhena, Poka, Ambon, 97233 Maluku, Indonesia; 3 Biology Centre, Academy of Sciences of the Czech Republic, Branisovska 31, Ceske Budejovice, Czech Republic; 4 Faculty of Agriculture, Gadjah Mada University, Jalan Bulaksumur, Yogyakarta, 55281, Indonesia

**Keywords:** Moluccas, Indo-Australia, Melanesia, Indonesia, Wallacea, species distributions, biogeography, taxonomy, habitat preferences, invasive species, biodiversity

## Abstract

The present checklist of ants (Hymenoptera: Formicidae) of Ambon is the first comprehensive overview of ant species recorded on the island during the last 150 years. The species list is based on literature and museum collections’ records combined with data from our field survey in 2010. In total, there are 74 ant species and subspecies representing 34 genera and six subfamilies known from Ambon. Five of the species found in undisturbed forest were exotic and indicate the overall habitat degradation on the island. The largest proportion of Ambon ant fauna are species with affinities to the Oriental region and species of Oriental-Austro-Melanesian origin. At least 20% of the species are regional endemics. In comparison to other islands in the region, the Ambon fauna seems more diverse and better sampled; however it is clear that a large part of it still remains to be described.

## Introduction

The Indonesian region of Moluccas (Maluku) is one of the most biogeographically and biologically complex areas on Earth. Many of its local invertebrate groups remain undescribed, including ant diversity, which is mostly unknown from this region. Despite being a frequent subject of ecological and taxonomic studies in surrounding regions, there are a limited number of ant species records from Moluccas (e.g. Chapman and Capco 1951, Trainor and Andersen 2010, Andersen et al. 2013).

Ambon is a smaller (775 km^2^), densely populated island (estimate for 2014 is 369 000 inhabitants; BPS-Statistics, Indonesia 2014) at the north of Banda islands arc, adjacent to the much larger Seram to the north. Ambon consists of two parts, Latimor and Hitu, which are connected by a short isthmus. Both parts are characterized by cordierite-rich dacites, granites, ultramafic rocks, and uplifted Quaternary coral reefs (Pownall et al. 2013). There are also two inactive volcanoes present on Hitu.

Ambon and Seram are located in the eastern Indonesia region, which is a geologically complex zone, influenced by the activity of three major plate regions: the Eurasian, Indian-Australian and Pacific-Philippine Sea plates. Boundaries among them form deformation zones that contain many small plates, crustal fragments or terranes and are characterized by the presence of subduction zones (Hall and Wilson 2000). The Banda Arc consists of an inner volcanic arc and an outer non-volcanic arc of islands formed by sedimentary, metamorphic and igneous rocks of Permian and Quaternary age (Hall 2000). Both Ambon and Seram lay within a belt of subducted Australian crust that is south of a deep subduction trench encircling the Banda Sea. Similar to most of the Banda Sea islands, Ambon and Seram are true oceanic islands, having never been connected to the mainland, although the distances between them and the Asian and Australian continents decreased significantly during periods of glaciation (Voris 2000). It is estimated that Ambon and Seram emerged above the sea about six to five million years ago (Fortuin and De Smet 1991, de Jong 1998).

Due to its position between the Oriental and Australian regions, the Moluccas host an interesting mixture of fauna and flora from both biogeographic origins, in addition to an important proportion of local endemics. Which faunal elements prevail is often taxon and island specific and has been a subject of multiple debates (Gressit 1958, Keast and Miller 1996). The distance between New Guinea and Ambon (and Seram) is shorter (140 km) then between Sulawesi (550 km). However, there are several islands between Ambon and Sulawesi which could serve as stepping stones. We can also expect that the exchange of biotas was mostly unidirectional as Ambon, Seram and other islands are younger than New Guinea and Sulawesi (de Jong 1998).

Based on the distribution of butterfly species, Vane-Wright and Peggie (1994) suggest that fauna of northern Moluccan islands (Halmahera, Ternate, Morotai and others) is biologically related to New Guinea, while the Buru, Ambon, Seram arc is related to North-West Australia and the Sula islands in west Moluccas are part of the ‘Sulawesi region’. A similar pattern was confirmed for the butterfly genus, *Hypolycaena* by Cassidy and Rawlins (2011), although the authors also found links among Ambon, Seram and Sulawesi as well as Mindanao in the Oriental zone. Close relationship between Moluccan and Sulawesi species was also suggested by phylogenetic reconstruction of the grasshopper genus *Chitaura* (Acrididae: Oxyinae) (Butlin et al. 1998).

A distributional analysis of hawk moths (Lepidoptera: Sphingidae) revealed that Ambon species are nested within Seram fauna and both are close to Halmahera and Sulawesi. They all are however less similar to south Philippines fauna than to New Guinean fauna (Beck and Kitching 1998). Finally, a comprehensive comparison based on the distribution of over 200 Lepidopteran species (de Jong 1998) supports greater similarity of Moluccan islands to New Guinea than to Sulawesi, suggesting a much higher diversity of New Guinean fauna and shorter distance as the main contributing factors. This pattern is also repeated at the generic level (de Jong 1998). These studies suggest a complex pattern of relationships among Mollucan Islands and the surrounding regions, with the most prominent links to Melanesia or Oriental regions (often through Sulawesi).

Unfortunately, no comparable data exist for Moluccan ant fauna. Although a number of species were described early on from specimens collected by A. R. Wallace (Smith 1859, 1860, 1871) and the regional list was expanded in the following 150 years, a detailed overview of the regional fauna is missing. In this respect, the most relevant studies are by Karavaiev (1925, 1927, 1929, 1930, 1935) and by Chapman and Capco (1951) which provide summaries of ant species from the Oriental and Austro-Melanesian regions.

In recent years, studies focusing on the ants of Timor Island were conducted, contributing important knowledge of ants of the region (Trainor at al. 2010; Andersen et al. 2013). In Timor, the largest proportion of ant species (76%) had South-East Asian (Oriental) affinities, while Australian taxa accounted for 18% of the fauna and the proportion was even smaller for other neighboring islands. In addition, a distributional analysis of ants of genus *Polyrhachis* confirms that at least some other Moluccan islands have high levels of endemism, even higher than plants and birds (Andersen et al. 2013). However, it is unclear to what extent these patterns are representative of northern Moluccan islands, as they are positioned closer to New Guinea which contributes Austro-Melanesian taxa, as well as to Sulawesi, which could serve as a source of Oriental fauna. Understanding the distribution patterns and origin of the ant fauna of Moluccas is desirable, since ants can provide another line of evidence for reconstructing the biogeographic history of the region. Due to their diverse biological roles, ants are ideal for assessing the effects of life strategy on dispersal and diversification patterns across oceanic islands.

Here, we assemble all available information on ants occurring on Ambon Island and combine it with new data from a recent field survey. The main objectives of our study are 1) to create a checklist which can be used as a reference for follow up studies; 2) to identify new species records collected during our survey of Sirimau forest in Ambon; and 3) to compare habitat preferences of collected ants, analyze biogeographical affinities, and asses the levels of endemism of ant fauna currently known from Ambon.

## Methods

### Survey of Sirimau area

We surveyed ants in forested areas of Sirimau, one of two protected areas of Ambon Island. Sirimau forest covers an area of approximately 3.5 Ha and is located on the eastern side of Ambon Island between -3.6707°; -3.6918° and 128.2394°; 128.2859°, at elevations between 100 to 200 meters above sea level. Although the study area has been referred to as ‘primary’ forest, it is more appropriate to call it ‘undisturbed’, as many parts of Ambon underwent habitat transformation in the last two centuries and a large proportion of the island’s surface was altered at some point. Today, urban areas cover more than 40% of the island (Miller 1999). The undisturbed forest at the collecting site was dominated by the following plant taxa: *Eucalyptus* sp., *Acacia mangium* (Willdenow, 1806), *Tectona grandis* (Linnaeus, 1782), *Nephelium lappaceum* (Linnaeus), *Anacardium occidentale* (Linnaeus), and *Shorea* sp.

Ants were collected in 500 m long transects each of which included 25 pitfall traps, 25 bait traps on the ground and 25 bait traps on vegetation. A mixture of sardines and honey was used for bait. The pitfall traps were exposed for two days and bait traps for one hour. Six such transects, located at least 200 m apart, were surveyed in undisturbed forest and six transects in secondary forest. In addition to collections in transects the area was repeatedly surveyed with general hand collecting and species not recorded in transects were added to the checklist. Samples were collected between July and September 2010 and preserved in 95% ethanol.

### Species identification and compilation of species list

Collected specimens were initially sorted to morphospecies, mounted and then identified by the authors using several reference collections: Basic Entomology Laboratory, Agriculture Faculty Gajah Mada University Jogyakarta, Indonesia; Ant Collection at Museum of Comparative Zoology, Harvard University, USA (MCZ); Melanesian Ants Collection at Czech Academy of Sciences (CAS); and taxonomic literature for relevant genera. For selected species which were not possible to match with museum, literature or online records, we sequenced ~ 650 bp fragment of mitochondrial gene cytochrome c oxidase (COI), using primers LCO1490 and HCO2198 (Folmer et al. 1994). The COI sequences were used to confirm species identities among samples and to improve the identification of unknown species. The sequences were compared with records available at the online databases, Barcodes of Life – BOLD (http://barcodinglife.org) and GenBank (www.ncbi.org), and used to identify the most closely related described species or species group and to confirm morphology-based identification. When we were not able to reliably match the specimen with a described species we used a morphospecies number and attempted to identify it to at least the closest described species or species group. The specimen duplicates were deposited in CAS, Czech Republic and the MCZ, USA. The DNA sequences of COI were deposited in the Barcode of Life database (http://barcodinglife.org), as part of the Ants of New Guinea project (ASPNA) and photos of selected specimens are available online at Ants of New Guinea database (www.newguineants.org, search query ‘Ambon’). Sequences were deposited in BOLD under accession numbers: ASPNA3025-14 – ASPNA3033-14.

The species checklist was compiled from collected specimens, literature records and database records. We reviewed the names of all taxa that were originally described from Ambon and included specimen records found on Antwiki (www.antwiki.org) and Antweb (www.antweb.org). Other resources including Formis 2013 (Porter and Wojcik 2013), Google Scholar (www.scholar.google.com) and Web of Science (www.webofknowledge.com) were also searched for relevant records (Ambon, Amboina etc.). We also searched for ant records associated with Seram Island and checked if species were recorded on Ambon as well. Searching the Indo-Australian collections deposited at the MCZ did not result in additional species records. We used the taxonomic nomenclature based on the online version of An Online Catalogue of the Ants of the World (Bolton 2014), as published at online Ant Catalogue (www.antcat.org). We eliminated names where the records could not be confirmed due to possible misidentification or erroneous and insufficient labeling. We retained trinomial names for all cases where status of the names were not disputed and considered valid.

### Data analysis

The specimen records from all traps and transects within the same habitat type were combined and the occurrence frequency of each species was used as input data. Diversity statistics and accumulation curves were calculated in EstimateS (Colwell 2013), using 100 iterations. To compare species diversity, evenness and species occurrence frequency between habitats we used the Shannon diversity index, Shannon diversity t-test and Mann-Whitney test in the program PAST 2.14 (Hammer et al. 2001).

For each described species in the checklist, we assembled data about its distribution range using online resources (Antwiki.org, Antweb.org) and the literature. The distribution categories reflected main areas of occurrence of the given taxa and were based on recognized biogeographic regions (Holt et al. 2013) with Oceania more finely divided into regions of Melanesia, Micronesia and Polynesia to provide more sensitivity for distributional comparisons. A species was considered widespread if it occurred in more than five major biogeographic regions. A species was considered introduced if it was known as non-native in the area or unlikely originating in Moluccas. A species was considered endemic it if was recorded only from Ambon or other Moluccan islands. This latter category was broader as the ant fauna of the whole region is highly undersampled and unknown. It is thus possible that species described from a particular island are in fact distributed across several neighboring islands. For these reasons our endemic species category included regional endemics.

## Results

Based on our literature survey, 48 species have been reported to occur on Ambon. In our field survey, we collected 31 ant species, 16 of which could be identified, while 15 did not match any described taxa on the basis of morphological or molecular characters. Of the 16 described species, only 5 species were previously reported. Our results suggest there are at least 74 ant species from 34 genera and six subfamilies on Ambon Island. The combined checklist of described ant species currently known from the island is 59.

**Figure 1.**
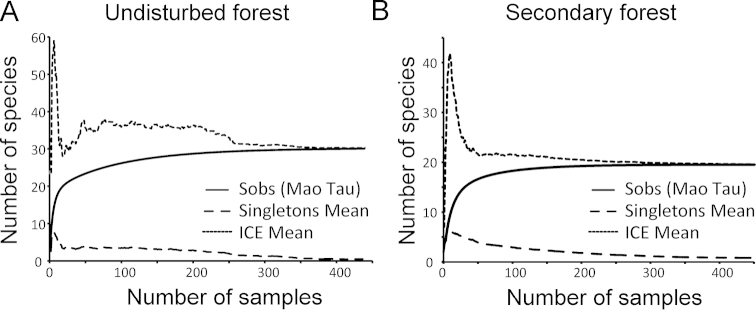
The cumulative number of species (Sobs) for 450 samples collected at both habitat types: **A** undisturbed forest **B** secondary forest. Each curve represents the average of 1000 replicate randomizations of samples order. Richness estimator ICE (mean) and average number of singletons are shown with dashed lines.

Habitat types differed in species richness: 30 ant species were found in undisturbed forest, while 19 ant species were found in secondary forest. All species occurring in secondary forest were also recorded in undisturbed forest; the overlap as expressed by Jaccard Index was 0.63. Shannon index of diversity for undisturbed forest was 2.96, and 2.6 for secondary forest. The habitats differed significantly in species occurrences, calculated as the proportion of visited traps (Shannon diversity t-test p < 0.01, t = 10.9, d.f. = 1010.5). The accumulation curves suggest that both habitats were sufficiently sampled, as both curves and the statistical indicators (Mao Tau, ICE) reached an asymptote (Figure 1).

The most frequent species in both undisturbed and disturbed forest was *Pheidole* sp. AMB01 (cf. *Pheidole submonticola* (Eguchi, 2001) from Borneo). The other most frequent species in undisturbed forest were *Pheidole* sp. AMB02, *Polyrhachis dives* (Smith, F., 1857), *Oecophylla smaragdina* (Fabricius, 1775), *Dolichoderus thoracicus* (Smith, F., 1860) and *Carebara* sp. AMB01 (cf. *Carebara diversa* (Jerdon, 1851)). In secondary forest plots, the most frequently encountered were *Anoplolepis gracilipes* (Smith, F., 1857) *Lophomyrmex opaciceps* (Viehmeyer, 1922), *Monomorium pharaonis* (Linnaeus, 1758) and *Odontoponera denticulata* (Smith, F., 1858). Such species composition is typical for secondary and disturbed forest vegetation in the Indo-Australian region.

**Figure 2.**
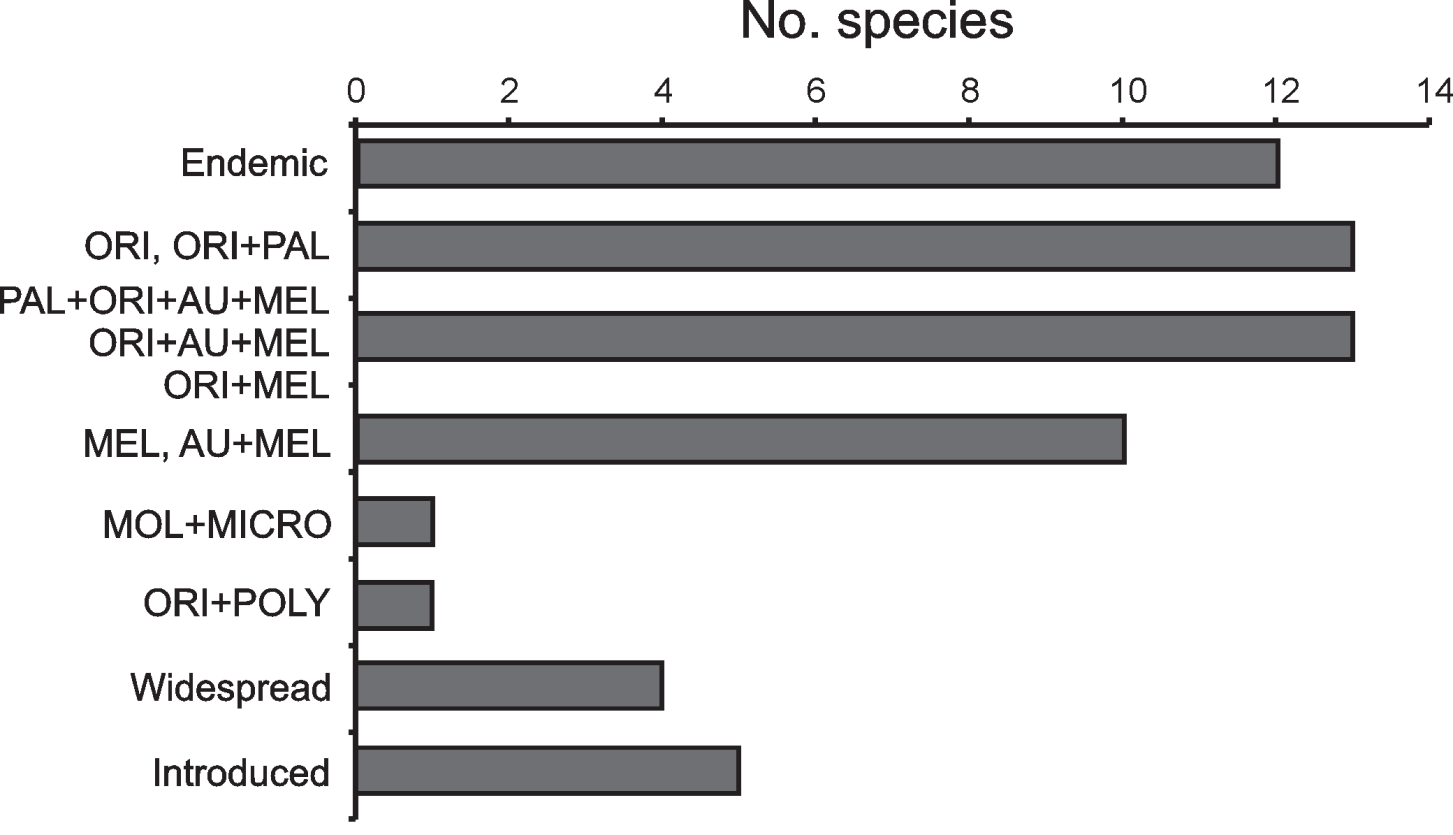
Biogeographic associations of valid ant species occurring on Ambon Island. The distribution categories reflected main areas of occurrence (see text). The abbreviations refer to following biogeographic regions and their combinations: AU – Australia, MEL – Melanesia, MIC – Micronesia, MOL – Moluccas, ORI – Oriental region, PAL – Palearctic region, POLY – Polynesia.

The comparisons of biogeographic associations of described species suggest that the ant fauna of Ambon is dominated by species with Oriental affinities (13 spp., 22%) and by species distributed equally across Oriental and Austro-Melanesian regions (13 spp., Figure 2). The proportion of species of solely Melanesian origin is slightly smaller (10 spp.) and there are 12 species (20%) which can be considered as local or regional endemics (restricted to Ambon and/or Moluccas). In addition, we detected five species which are most likely introduced on the island. A smaller proportion of species are either widespread (occurring in five or more biogeographic regions) or have more restricted distribution throughout Moluccas (Figure 2).

## Discussion

Our study provides the first formal checklist for ant species occurring at Ambon Island. In addition to nearly 50 species reported previously from the literature and databases, our field survey contributed new distributional records for 11 species (marked in Table 1). The additional 15 species which did not match with any currently described taxa are likely to expand the checklist once incorporated into taxonomic revisions of corresponding groups. Our survey also confirms the presence of several species recorded more than 100 years ago and provides evidence of several introduced species in the area.

**Table 1.** List of valid species recorded from the Ambon Island arranged by subfamily, genus and species. Column ‘Habitat’ refers to habitat type at which species was recorded during this study, U = undisturbed forest; S = secondary forest. Abbreviations in ‘Distribution’ category refer to following biogeographic regions: AU – Australia, MEL – Melanesia, MIC – Micronesia, ORI – Oriental region, PAL – Palearctic region, POLY – Polynesia, Intro. – introduced species, Wide – widespread species.

Taxon	Author	Distribution	Habitat (U/S)
Amblyoponinae			
*Myopopone castanea*	(Smith F, 1860)	ORI, AU, MEL	
Dolichoderinae			
*Dolichoderus thoracicus*	(Smith F, 1860)	ORI	U, S
*Iridomyrmex anceps*	Roger, 1863	Wide	U, S
*Iridomyrmex pallidus*	Forel, 1901	AU, MEL	
*Tapinoma melanocephalum*	Fabricius, 1793	Intro.	U, S
Formicinae			
*Acropyga acutiventris*	Roger, 1862	ORI,AU,MEL	
*Anoplolepis gracilipes*	(Smith F, 1857)	Intro.	U, S
*Camponotus bellus*	Forel, 1908	ORI	
*Camponotus quadriceps*	(Smith F, 1859)	MEL	
*Camponotus indefinitus*	Karavaiev, 1929	Ambon	
*Camponotus variegatus ambonensis*	Karavaiev, 1930	Ambon	
*Echinopla striata gibbosa*	Karavaiev, 1927	ORI	
*Oecophylla smaragdina*	(Fabricius, 1775)	ORI, AU, MEL	U, S
*Opisthopsis halmaherae*	Karavaiev, 1930	Moluccas	
*Paratrechina longicornis*	(Latreille, 1802)	Intro.	U, S
*Polyrhachis arcuata*	(Le Guillou, 1842)	ORI, AU	
*Polyrhachis dives*	Smith F, 1857	ORI, PAL, AU, MEL	U, S
*Polyrhachis fervens*	Smith F, 1860	ORI, AU, MEL	
*Polyrhachis pressa*	Mayr, 1862	ORI	U
*Polyrhachis batesi*	Forel, 1911	Ambon, Seram	
*Polyrhachis bicolor weyeri*	Karavaiev, 1930	Ambon	
*Polyrhachis creusa*	Emery, 1897	ORI, AU, MEL	
*Polyrhachis denticulata*	Karavaiev, 1927	AU,MEL	
*Polyrhachis dives rectispina*	Karavaiev, 1927	Ambon	
*Polyrhachis derecyna*	Smith F, 1871	MEL	
*Polyrhachis dolomedes*	Smith F, 1871	ORI, MEL	
*Polyrhachis fervens*	Smith F, 1860	ORI, AU, MEL	
*Polyrhachis fruhstorferi torta*	Santschi, 1928	Ambon	
*Polyrhachis goramensis*	Emery, 1887	Ambon	
*Polyrhachis keratifera*	Karavaiev, 1927	Ambon	
*Polyrhachis nigrescens*	Karavaiev, 1927	Ambon	
*Polyrhachis radicicola*	Dahl, 1901	MEL	
*Polyrhachis rastellata*	(Latreille, 1802)	ORI, AU, MEL	
*Polyrhachis rufofemorata*	Smith F, 1859	AU, MEL	U, S
*Polyrhachis tibialis orientalis*	Karavaiev, 1927	Ambon	
*Polyrhachis tricuspis*	André, 1887	Moluccas	
*Polyrhachis vindex*	Smith F, 1857	ORI	
*Pseudolasius breviceps*	Emery, 1887	MEL	
*Pseudolasius familiaris*	(Smith F, 1860)	ORI	
*Pseudolasius mayri bedoti*	Emery, 1911	Ambon	
Myrmicinae			
*Calyptomyrmex beccarii*	Emery, 1887	ORI, AU, MEL	
*Cardiocondyla nigrocerea*	Karavaiev, 1935	Moluccas	
*Carebara weyeri*	Karavaiev, 1930	Ambon	
*Lophomyrmex opaciceps*	Viehmeyer, 1922	ORI	U, S
*Monomorium pharaonis*	(Linnaeus, 1758)	Intro.	U, S
*Pheidole ambonensis*	Karavaiev, 1930	Ambon	
*Pheidole plagiaria*	Smith F, 1860	ORI	
*Podomyrma basalis reyi*	Karavaiev, 1935	Ambon	
*Solenopsis geminata*	(Fabricius, 1804)	Intro.	U, S
*Strumigenys doriae*	Emery, 1887	ORI	
*Tetramorium pacificum*	Mayr, 1870	ORI, AU, MEL	
Ponerinae			
*Anochetus graeffei*	Mayr, 1870	Wide	U
*Diacamma rugosum*	(Le Guillou, 1842)	ORI, MEL	
*Odontomachus saevissimus*	Smith F, 1858	MEL, Moluccas	
*Odontomachus simillimus*	Smith F, 1858	Wide	U
*Odontoponera denticulata*	(Smith F, 1858)	ORI	U
*Brachyponera atrata*	Karavaiev, 1925	Moluccas, MIC	
*Ectomomyrmex striatulus*	Karavaiev, 1935	AU, MEL	
*Platythyrea parallela*	Smith F, 1859	Wide	U

**Table 2.** List of unidentified and undescribed species collected during our survey of Sirimau forest, Ambon. U = undisturbed forest; S = secondary forest.

Taxon	Ident. note	Habitat	Voucher code
Ectatomminae			
*Rhytidoponera* sp. AMB01		U, S	FLAM10/0125
Formicinae			
*Camponotus* sp. AMB01		U, S	FLAM10/0073
*Camponotus* sp. AMB02		U	FLAM10/0001
*Camponotus* sp. AMB03		U	FLAM10/0040
*Nylanderia* sp. AMB01	cf. *Nylanderia vividula*	U, S	FLAM10/0013
*Polyrhachis* sp. AMB04		U, S	FLAM10/0121
Myrmicinae			
*Crematogaster* sp. AMB01		U	FLAM10/0044
*Pheidole* sp. AMB01	cf. *Pheidole submonticola*	U, S	FLAM10/0051
*Pheidole* sp. AMB02	cf. *Pheidole submonticola*	U	FLAM10/0037
*Pheidole* sp. AMB03	*Pheidole umbonata* group	U	FLAM10/0027
*Pheidole* sp. AMB04		U	FLAM10/0026
*Carebara* sp. AMB01	cf. *Carebara diversa*	U, S	FLAM10/0018
*Romblonella* sp. AMB01		U	FLAM10/0012
Ponerinae			
*Diacamma* sp. AMB01		U, S	FLAM10/0141
*Leptogenys* sp. AMB01		U, S	FLAM10/0125A

Despite the addition of 26 species to the checklist, the total number of ant species recorded from Ambon (74) is most likely underestimated. Our survey focused on forested area of only one of the two Ambon islands and in the current checklist some of the genera (e.g. *Pheidole*, *Tetramorium*, *Camponotus*, *Nylanderia*) which are generally diverse in this region are represented by only few species or completely missing. Our study also did not include sampling of more cryptic leaf litter fauna which would probably contribute substantially to the local species list. For example, we anticipate that species diversity of the genera *Pheidole*, *Nylanderia*, *Hypoponera* and *Strumigenys* will be higher than reported here. On the other hand, we report significantly higher species richness than the number of species recorded from the southern Moluccan islands of Wetar (3600 km^2^), Pantar (728 km^2^) and Lembata (Trainor and Andersen 2010) or the Solomon Islands (e. g. Lembata – 70 ant spp., Sarnat et al. 2013) which are larger than or of similar size to Ambon. We anticipate that the ant species richness of Ambon might be even higher than that of other comparably sized islands, as it is adjacent to the much larger Seram, which could potentially serve as a source area for ant species that would otherwise go extinct on small and disturbed islands.

The accumulation curves indicate that both our sites were sampled exhaustively, within the limits of the methods used. This suggests that our survey provides a representative view of the above ground foraging ant fauna of the area. General collecting in the area outside the transects yielded only one additional species (*Anochetus graeffei* Mayr, 1870). The fact that all the species from secondary forest were also found in undisturbed forest is somewhat surprising and probably indicates a limited regional species pool and relative similarity of both types of habitats. A thorough sampling of other microhabitats (leaf litter, canopy) could contribute additional species.

Both forests were also inhabited by several invasive species not native to the area, including: *Anoplolepis gracilipes*, *Paratrechina longicornis* (Latreille, 1802), *Solenopsis geminata* (Fabricius, 1804) and *Monomorium pharaonis*. Although, these species mostly did not reach high frequencies in either type of forest (only *Anoplolepis gracilipes* reached a high frequency, being the fourth most frequent species in disturbed forest), their presence is indicative of medium to strong habitat disturbance (Invasive Species Specialist Group, www.issg.org, New Zealand biosecurity system, http://www.biosecurity.govt.nz). The most frequently collected taxa in both habitats were several species of *Pheidole* and *Polyrhachis*, as well as *Oecophylla smaragdina* and *Dolichoderus thoracicus*.

Although the undisturbed forest site was inhabited by 11 more species than the disturbed site, the high abundances of *Oecophylla smaragdina*, *Dolichoderus thoracicus* and moderate abundances of *Monomorium pharaonis* and *Tapinoma melanocephalum* (Fabricius, 1793) indicate that this area has likely been disturbed in the past, as these species are often associated with secondary or disturbed forests (Invasive Species Specialist Group database, http://www.issg.org, Way and Khoo 1991, Klimes et al. 2012).

### Biogeographic affinities

Out of the 59 valid species currently known from Ambon, the largest proportion (13 spp.) is of Oriental (South East Asian) and Oriental-Austro-Melanesian origin (13 spp.). The latter category represents species which are equally distributed across Oriental and Austro-Melanesian regions, mostly spread across islands of South East Asia, New Guinea and/or Australia. The third largest group of Ambon myrmecofauna is of local origin (12 spp.). Here, they represent a smaller proportion of fauna compared to data from the ant genus *Polyrhachis* on the much larger Timor (~70% endemics, Andersen et al. 2013). Our data suggest that the Oriental faunal elements are somewhat more prominent in Ambon than Austro-Melanesian elements. The most important part of the fauna are however regional endemics and species distributed equally across parts of the Oriental and Austro-Melanesian regions – an area which corresponds with a largely extended Wallacea. This group includes taxa that either originated within this region and/or lineages which expanded into the region from either Oriental or Australian continental shelves. However, without detailed phylogenetic reconstruction for each of these genera it is not possible to reveal their exact area of origin. A detection of five introduced species in a relatively preserved habitat should be of conservational concern and suggests that these exotic species are well established in Ambon. This is indeed a case in other parts of the island, especially in urban and suburban areas (FL personal observation).

## Conclusions

Our field survey and literature search revealed that there are over 70 ant species and subspecies found on Ambon Island, of which at least 59 have been previously described. The total number is likely to increase once data from other parts of the island and from understudied microhabitats such as tree canopy or forest leaf litter become available. The occurrence of several non-native ant species recorded in old preserved forest is of conservational concern and suggests that the introduced species are well established on the island. The overall structure of the ant community recorded in the forested area is indicative of a strong effect of habitat fragmentation and large habitat conversion on the island, where urban habitat is prominent. From a biogeographic perspective, the largest portions of Ambon ant fauna are species with Oriental affinities and species native to the Oriental-Austro-Melanesian region. Interestingly, the portion of Austro-Melanesian species is smaller which contrasts to patterns observed in Moluccan Lepidoptera. Our study provides the first basis for future research on ants of Ambon Island and surrounding areas. As habitat conversion in this region continues, the unique and endemic fauna of Moluccas is particularly endangered and in urgent need of proper conservational efforts. Ants, along with moths and butterflies can serve as useful indicators of habitat quality and uniqueness and for identification of conservational priorities.

## References

[B1] Andersen AN Kohout RJ Trainor CR (2013) Biogeography of Timor and surrounding Wallacean Islands: endemism in ants of the genus *Polyrhachis* Fr. Smith. Diversity 5: 139–148. doi: 10.3390/d5010139

[B2] André E (1887) Description de quelques fourmis nouvelles ou imparfaitement connues. Revue d’Entomologie (Caen) 6: 280–298.

[B3] Beck J Kitching IJ (2008) The Sphingidae of Southeast-Asia version 1.5. http://www.sphin-sea.unibas.ch/SphinSEA/SphinSEA_home.htm

[B4] Bolton B (2014) An online catalogue of the ants of the world. http://antcat.org

[B5] BPS-Statistics Indonesia (2014) Statistics Indonesia. http://maluku.bps.go.id/

[B6] Butlin RK Walton CW Monk KA Bridle JR (1998) Biogeography of Sulawesi grasshoppers, genus *Chitaura*, using DNA sequence data. In: Hall R Holloway JD Rosen BR (Eds) Biogeography and geological evolution of South-East Asia. Backhuys Publishers, 355–359.

[B7] Cassidy A Rawlins A (2011) On Hypolycaena from Maluku, Indonesia, including the first description of male *Hypolycaena asahi* (Lepidoptera, Lycaenidae). Zookeys 115: 53–84. doi: 10.3897/zookeys.115.1406 21977001 10.3897/zookeys.115.1406 PMC3187666

[B8] Chapman JW Capco SR (1951) Check list of the ants (Hymenoptera: Formicidae) of Asia. Monographs of the Institute of Science and Technology Manila 1: 1–327.

[B9] Colwell RK (2013) EstimateS, Statistical Estimation of Species Richness and Shared Species from Samples. http://viceroy.eeb.uconn.edu/estimates/

[B10] Dahl F (1901) Das Leben der Ameisen im Bismarck-Archipel, nach eigenen Beobachtungen vergleichend dargestellt. Mitteilungen aus dem Zoologischen Museum in Berlin 2: 1–70.

[B11] de Jong R (1998) Halmahera and Seram: Different histories, but similar butterfly faunas. In: Hall R Holloway JD Rosen BR (Eds) Biogeography and geological evolution of South-East Asia. Backhuys Publishers, 315–325.

[B12] Eguchi K (2001) A revision of the Bornean species of the ant genus Pheidole (Insecta: Hymenoptera: Formicidae: Myrmicinae). Tropics Monograph Series 2: 1–154.

[B13] Emery C (1887) Catalogo delle formiche esistenti nelle collezioni del Museo Civico di Genova. Parte terza. Formiche della regione Indo-Malese e dell’Australia. Annali del Museo Civico di Storia Naturale 24: 209–240.

[B14] Emery C (1897) Viaggio di Lamberto Loria nella Papuasia orientale. XVIII. Formiche raccolte nella Nuova Guinea dal Dott. Lamberto Loria. Dummy reference. Annali del Museo Civico di Storia Naturale 38: 546–594.

[B15] Emery C (1911) Fragments myrmécologiques. Annales de la Société Entomologique de Belgique 55: 213–225.

[B16] Fabricius JC (1775) Systema entomologiae, sistens insectorum classes, ordines, genera, species adiectis synonymis, locis, descriptionibus, observationibus. Korte, Flensburgi et Lipsiae, 832 pp.

[B17] Fabricius JC (1793) Entomologia systematica emendata et aucta. Secundum classes, ordines, genera, species, adjectis synonimis, locis observationibus, descriptionibus. Tome 2 C. G. Proft, Hafniae, 519 pp.

[B18] Fabricius JC (1804) Systema Piezatorum secundum ordines, genera, species, adjectis synonymis, locis, observationibus, descriptionibus. C. Reichard, Brunswick, xiv + 15–439.

[B19] Folmer O Black M Hoeh W Lutz R Vrijenhoek R (1994) DNA primers for amplification of mitochondrial cytochrome c oxidase subunit I from diverse metazoan invertebrates. Molecular Marine Biology and Biotechnology 3: 294–299. 7881515

[B20] Forel A (1901) Formiciden aus dem Bismarck-Archipel, auf Grundlage des von Prof. Dr. F. Dahl gesammelten Materials. Mitteilungen aus dem Zoologischen Museum in Berlin 2: 4–37.

[B21] Forel A (1908) Ameisen aus Sao Paulo (Brasilien), Paraguay etc. gesammelt von Prof. Herm. v. Ihering, Dr. Lutz, Dr. Fiebrig, etc. Verhandlungen der Kaiserlich-Königlichen Zoologisch-Botanischen Gesellschaft in Wien 58: 340–418.

[B22] Forel A (1911) Die Ameisen des K. Zoologischen Museums in München. Sitzungsberichte der Mathematischen-Physikalischen Klasse der Königlich Bayerischen Akademie der Wissenschaften zu München 11: 249–303.

[B23] Fortuin AR De Smet MEM (1991) Rates and magnitudes of late Cenozoic vertical movements in the Indonesian Banda Arc and the distinction of eustatic effects. In: Macdonald DIM (ed.) Sedimentation, Tectonics and Eustasy - Sea-level changes at active margins.Special Publication of the Iinternational Association of Sedimentologists 12: 79–89.

[B24] Gressitt JL (1958) Zoogeography of Insects. Annual Review of Entomology 3: 207–230. doi: 10.1146/annurev.en.03.010158.001231

[B25] Hall R Wilson MEJ (2000) Neogene sutures in eastern Indonesia. Journal of Asian Earth Sciences 18: 781–808. doi: 10.1016/S1367-9120(00)00040-7

[B26] Hammer O Harper DAT Ryan PD (2001) PAST: paleontological statistics software package for education and data analysis. Palaeontologia Electronica 4: Unpaginated.

[B27] Holt BG Lessard J-P Borregaard MK Fritz S Bastos Araujo M Dimitrov DS Fabre P-HF Graham CH Graves GR Jønsson KA Nogues DB Wang Z Whittaker RJ Fjeldså J Rahbek C (2013) An update of Wallace’s Zoogeographic Regions of the World. Science 339(6115): 74–78. doi: 10.1126/science.1228282 23258408 10.1126/science.1228282

[B28] Invasive Species Specialist Group (2014) http://www.issg.org

[B29] Jerdon TC (1851) A catalogue of the species of ants found in Southern India. Madras Journal of Literature and Science 17: 103–127.

[B30] Karavaiev V (1925) Ponerinen (Fam. Formicidae) aus dem Indo-Australischen Gebiet. (Fortsetzung). Konowia 4: 115–131.

[B31] Karavaiev V (1927) Ameisen aus dem Indo-Australischen Gebiet. III. Zbirnyk Prats’ Zoolohichnoho Muzeyu 3: 3–52 [=Trudy. Ukrains’ka Akademiya Nauk. Fizichno-Matematichnoho Viddilu 57: 53–52]

[B32] Karavaiev V (1929) Ameisen aus dem Indo-Australischen Gebiet. VI. Zbirnyk Prats’ Zoolohichnoho Muzeyu 7: 235–248 [=Trudy. Ukrains’ka Akademiya Nauk. Fizichno-Matematichnoho Viddilu 213: 233–246]

[B33] Karavaiev V (1930) Ameisen von den Molukken und Neuguinea. (Ergebnisse der Sunda-Expedition der Notgemeinschaft der deutschen Wissenschaft 1929/30.). Zoologischer Anzeiger 92: 206–214.

[B34] Karavaiev V (1935) Neue Ameisen aus dem Indo-Australischen Gebiet, nebst Revision einiger Formen. Treubia 15: 57–118.

[B35] Keast A Miller SE (1996) The origin and evolution of Pacific island biotas, New Guinea to eastern Polynesia: patterns and processes. SPB Academic Publishing, Amsterdam, The Netherlands, 531 pp.

[B36] Klimes P Idigel C Rimandai M Fayle TM Janda M Weiblen GD Novotny V (2012) Why are there more arboreal ant species in primary than in secondary tropical forests? Journal of Animal Ecology 81(5): 1103–1112. doi: 10.1111/j.1365-2656.2012.02002.x 22642689 10.1111/j.1365-2656.2012.02002.x

[B37] Latreille PA (1802) Histoire naturelle des fourmis, et recueil de mémoires et d’observations sur les abeilles, les araignées, les faucheurs, et autres insectes. Impr. Crapelet (chez T. Barrois), Paris, xvi + 445 pp.

[B38] Le Guillou EJF (1842) Catalogue raisonné des insectes hyménoptères recueillis dans le voyage de circumnavigation des corvettes l’Astrolabe et la Zélée. Annales de la Société Entomologique de France 10: 311–324.

[B39] Linnaeus C (1758) Systema naturae per regna tria naturae, secundum classes, ordines, genera, species, cum characteribus, differentiis, synonymis, locis. Tomus I Editio decima, reformata L. Salvii, Holmiae, 824 pp.

[B40] Linnaeus C (1782) Supplementum Plantarum. Impensis Orphanotrophei, Brunsvigae, 151.

[B41] Mayr G (1862) Myrmecologische Studien. Verhandlungen der Kaiserlich-Königlichen Zoologisch-Botanischen Gesellschaft in Wien 12: 649–776.

[B42] Mayr G (1870) Neue Formiciden. Verhandlungen der Kaiserlich-Königlichen Zoologisch-Botanischen Gesellschaft in Wien 20: 939–996.

[B43] Miller A (1999) Resource Management in the Urban Sphere: Ambon’s Urban Environment. Cakalele 10: 7–37.

[B44] New Zealand biosecurity system (2014) http://www.biosecurity.govt.nz

[B45] Pownall JM Hall R Watkinson I (2013) Extreme extension across Seram and Ambon, eastern Indonesia: evidence for Banda slab rollback. Solid Earth 4: 277–314. doi: 10.5194/se-4-277-2013

[B46] Porter S Wojcik D (2013) Formis 2013, A Master Bibliography of Ant Literature. http://www.ars.usda.gov/News/docs.htm?docid=10003&page=3

[B47] Roger J (1862) Einige neue exotische Ameisen-Gattungen und Arten. Berliner Entomologische Zeitschrift 6: 233–254.

[B48] Roger J (1863) Die neu aufgeführten Gattungen und Arten meines Formiciden-Verzeichnisses nebst Ergänzung einiger früher gegebenen Beschreibungen. Berliner Entomologische Zeitschrift 7: 131–214. doi: 10.1002/mmnd.18630070116

[B49] Santschi F (1928) Fourmis de Sumatra, récoltées par Mr. J. B. Corporaal. Tijdschrift voor entomologie 71: 119–140.

[B50] Sarnat EM Blanchard B Guenard B Fasi J Economo EP (2013) Checklist of the ants (Hymenoptera, Formicidae) of the Solomon Islands and a new survey of Makira Island. Zookeys 257: 47–88. doi: 10.3897/zookeys.257.4156 23653494 10.3897/zookeys.257.4156 PMC3591739

[B51] Smith F (1857) Catalogue of the hymenopterous insects collected at Sarawak, Borneo; Mount Ophir, Malacca; and at Singapore, by A. R. Wallace. Journal and Proceedings of the Linnean Society of London. Zoology 2: 42–88.

[B52] Smith F (1858) Catalogue of hymenopterous insects in the collection of the British Museum. Part VI. Formicidae. British Museum, London, 216 pp.

[B53] Smith F (1859) Catalogue of hymenopterous insects collected by Mr. A. R. Wallace at the islands of Aru and Key. [part]. Journal of the proceedings of the Linnean Society of London. Zoology 3: 132–158.

[B54] Smith F (1860) Catalogue of hymenopterous insects collected by Mr. A. R. Wallace in the islands of Bachian, Kaisaa, Amboyna, Gilolo, and at Dory in New Guinea. Journal of the proceedings of the Linnean Society of London. Zoology 5: 93–143.

[B55] Smith F (1871) A catalogue of the Aculeate Hymenoptera and Ichneumonidae of India and the Eastern Archipelago. With introductory remarks by A. R. Wallace. [part]. Journal of the proceedings of the Linnean Society of London. Zoology 11: 285–348.

[B56] Trainor CR Andersen AN (2010) The ant fauna of Timor and neighbouring islands: potential bridges between the disjunct faunas of South East Asia and Australia. Australian Journal of Zoology 58: 133–144. doi: 10.1071/ZO09113

[B57] Vane-Wright RI Peggie D (1994) The butterflies of northern and central Maluku: diversity, endemism, biogeography, and conservation priorities. Tropical Biodiversity 2: 212–230.

[B58] Viehmeyer H (1922) Neue Ameisen. Archiv für Naturgeschichte 88: 203–220.

[B59] Voris HK (2000) Maps of Pleistocene sea levels in Southeast Asia: shorelines, river systems and time durations. Journal of Biogeography 27: 1153–1167. doi: 10.1046/j.1365-2699.2000.00489.x

[B60] Way MJ Khoo KC (1991) Colony dispersion and nesting habits of the ants, *Dolichoderus thoracicus* and *Oecophylla smaragdina* (Hymenoptera: Formicidae) in relation to their success as biological control agents on cocoa. Bulletin of Entomological Research 81: 341–350. doi: 10.1017/S0007485300033629

[B61] Willdenow CL (1806) Species Plantarum. Editio quarta 4: 1053–1054.

